# The Next Generation Scientist program: capacity-building for future scientific leaders in low- and middle-income countries

**DOI:** 10.1186/s12909-018-1331-y

**Published:** 2018-10-10

**Authors:** Goonaseelan Pillai, Kelly Chibale, Edwin C. Constable, Akiko N. Keller, Marcelo M. Gutierrez, Fareed Mirza, Christian Sengstag, Collen Masimirembwa, Paolo Denti, Gary Maartens, Michèle Ramsay, Bernhards Ogutu, Eyasu Makonnen, Richard Gordon, Carlos Gil Ferreira, Fernando Alberto Goldbaum, Wim M. S. Degrave, Jonathan Spector, Brigitta Tadmor, Hedwig J. Kaiser

**Affiliations:** 1 CP+ Associates GmbH, Basel, Switzerland; 2 Division of Clinical Pharmacology, Department of Medicine, University of Cape Town, Cape Town, South Africa; 3 Drug Discovery and Development Centre (H3D), South African Medical Research Council Drug Discovery and Development Research Unit and Institute of Infectious Disease and Molecular Medicine, University of Cape Town, Cape Town, South Africa; 4 University of Basel, Basel, Switzerland; 5 Novartis, Basel, Switzerland; 6 African Institute of Biomedical Science & Technology, Harare, Zimbabwe; 7 Sydney Brenner Institute for Molecular Bioscience and Division of Human Genetics, Faculty of Health Sciences, University of the Witwatersrand, Johannesburg, South Africa; 8 University of Strathmore and Kenya Medical Research Institute, Nairobi, Kenya; 9 Center For Innovative Drug Development and Therapeutic Trials for Africa, College of Health Sciences, Addis Ababa University, Addis Ababa, Ethiopia; 10 Medical Research Council, Cape Town, South Africa; 11 D’or Institute for Research and Education, Rio de Janeiro, Brazil; 12 Leloir Institute Foundation, Buenos Aires, Argentina; 13 Oswaldo Cruz Institute, Fiocruz, Rio de Janeiro, Brazil; 14 Novartis, Cambridge, USA

**Keywords:** Capacity development, Capability development, Research and Development, Public health, Education, Fellowship, Postgraduate research, Early career researcher development

## Abstract

**Background:**

Scientific and professional development opportunities for early career scientists in low- and middle- income countries (LMICs) are limited and not consistent. There is a disproportionately low number of biomedical and clinical researchers in LMIC’s relative to their high burden of disease, a disparity that is aggravated by emigration of up to 70% of scientists from their countries of birth for education and employment elsewhere. To help address this need, a novel University-accredited, immersive fellowship program was established by a large public-academic-private network. We sought to describe the program and summarize progress and lessons learned over its first 7-years.

**Methods:**

Hallmarks of the program are a structured learning curriculum and bespoke research activities tailored to the needs of each fellow. Research projects expose the scientists to state-of-the-art methodologies and leading experts in their fields while also ensuring that learnings are implementable within their home infrastructure. Fellows run seminars on drug discovery and development that reinforce themes of scientific leadership and teamwork together with practical modules on addressing healthcare challenges within their local systems. Industry mentors achieve mutual learning to better understand healthcare needs in traditionally underserved settings. We evaluated the impact of the program through an online survey of participants and by assessing research output.

**Results:**

More than 140 scientists and clinicians from 25 countries participated over the 7-year period. Evaluation revealed strong evidence of knowledge and skills transfer, and beneficial self-reported impact on fellow’s research output and career trajectories. Examples of program impact included completion of post-graduate qualifications; establishment and implementation of good laboratory- and clinical- practice mechanisms; and becoming lead investigators in local programs. There was a high retention of fellows in their home countries (> 75%) and an enduring professional network among the fellows and their mentors.

**Conclusions:**

Our experience demonstrates an example for how multi-sectoral partners can contribute to scientific and professional development of researchers in LMICs and supports the idea that capacity-building efforts should be tailored to the specific needs of beneficiaries to be maximally effective. Lessons learned may be applied to the design and conduct of other programs to strengthen science ecosystems in LMICs.

**Electronic supplementary material:**

The online version of this article (10.1186/s12909-018-1331-y) contains supplementary material, which is available to authorized users.

## Background

Establishing sustainable research in science ecosystems in low- and middle-income countries (LMICs) is an urgent priority but no small task [1–3]. In these countries, the number of biomedical and clinical researchers is disproportionately low relative to the high burden of disease, a disparity that is aggravated by emigration of up to 70% of scientists from their countries of birth in pursuit of education and employment elsewhere [4–6]. To break this self-propagating cycle, scientific research infrastructures in LMICs will need to develop to a tipping point beyond which scientists have greater professional incentives to remain in their home countries. There have been major efforts to strengthen science systems in LMICs, including collaborations focused on capacity-building involving local research institutions and global stakeholders. These programs have met with various levels of success [7–11]. However, much more progress is needed.

Achievements in science and medicine that impact humanity in LMICs ultimately depend on communities of scientists and clinicians that live and work in those regions. Human capital development is an important component to improvement of science and medical capacity in LMICs [2, 3]. Local scientific training curricula that are contemporary and high-quality have expanded over the past decade [12, 13]. To maintain this momentum, comprehensive knowledge and skills training must continue and be expanded across a range of scientific disciplines.

In response to needs for enhanced professional development for scientists in LMICs, a novel fellowship training program has been implemented over the past 7 years by a network of public, academic and private partners. Target beneficiaries were Master’s, PhD, and post-doctoral scientists and clinicians who wished to pursue scientific and leadership skills development to complement instruction at their home institution. The program’s central theme was individualized mentoring, and research activities were tailored to the unique needs of each participant. The core program took place annually over a 3-month period in Switzerland, where fellows received on-site tutoring at Novartis’ research and development laboratories followed by accreditation from the University of Basel. Mentoring and collaboration often continued long after completion of the core program. The initiative was named the “Next Generation Scientist” (NGS) program to highlight its stated objective of contributing to the development of future scientific leaders in LMICs.

The NGS program recently graduated its 140th fellow. To our knowledge, there have been few capacity-building efforts designed specifically for scientists in LMICs, and still fewer that have attempted to assess their impact and successes [7]. We sought to summarize the progress and lessons learned and to evaluate outcomes including research output and influence on the participants’ subsequent career trajectories.

## Methods

### Program origin and governance

The NGS program was established in 2011 as part of a multi-faceted effort at Novartis to help build science capacity in LMICs [14–16], and to provide opportunities for industry scientists and clinicians to better understand medical needs in diverse, traditionally underserved groups [17]. The goal was to create an environment for mutual learning [18] through scientist-to-scientist interactions clustered around research activities tailored to the unique needs of each fellow. Since 2014, the program has been jointly coordinated with the University of Basel as a natural extension of the university’s long-standing work in LMICs and as a leading center in Switzerland for Africa-focused academic initiatives.

### Fellows and scientific mentors

Each year from 2011 to 2017, an open call for applications was disseminated through public channels. Applicants were entered into a rigorous selection process comprising a dossier review (curriculum vitae, description of research interests, and essay-style letters of motivation and support) and up to three rounds of interviews conducted by telephone or in person. Interviews were led by academic researchers, prospective scientific mentors, and trained human resource professionals. Key selection criteria included: academic credentials of the candidate (Master’s equivalent and above); ability to match applicants’ research interests and desired skills with an appropriate scientific mentor and host laboratory; and candidates’ long-term commitment to conduct biomedical or clinical research in their home country. The University of Basel and Novartis jointly approved the final cohort of candidates, which typically consisted of 20–25 fellows each year.

Scientific mentors at the University of Basel and Novartis participated in a volunteer capacity. Generally, this was because they wanted to engage in an academic-style mentoring program, were interested in the possibility of scientific collaborations with research institutions in LMICs, or were motivated by the opportunity to personally contribute to the advancement of science in LMICs. Mentors were selected based on their specialties and their anticipated ability to dedicate sufficient effort and be effective tutors. Many mentors chose to participate over several years. Mentors were also offered access to mentorship training programs and were provided with operational support from a small core team at University of Basel and Novartis responsible for management of the program. The mentors also participated in periodic meetings for peer support and sharing of mentorship best practices. Fellows and mentors jointly developed a research plan according to the fellow’s interests and skills.

### Program structure

The 3-month core program comprised two main components: a bespoke research project and a formally structured science and leadership skills development program. The research project occupied the fellow for approximately 80% of the time spent on-site and was designed to be sustainably implementable within infrastructure at the fellow’s home institution. Research interests of the selected candidates were identified from their application dossiers and matched with prospective mentors that had similar interests and relevant skillsets. The scope of work for each fellow was then iteratively refined through virtual (telephone and email) discussions between candidates and mentors. Whenever feasible, candidates’ home supervisors joined the pre-fellowship discussions to contribute recommendations for technical aspects that would benefit the fellow and the home institution. Once selected, mentors and mentees were typically in close contact leading up to commencement of the on-site stay (e.g. to exchange background materials; to attend to logistics of data and sample transfer; and to guide the candidate to learn basic skills in preparation for training that they would receive during the fellowship). Topics in the scientific curriculum were allocated to groups of fellows who planned and organized seminars which facilitated bi-directional interactions with senior experts. The leadership skills program comprised workshops focused on written and oral scientific communication, self-awareness, and appreciative inquiry [19]. Workshops were highly interactive, immersive experiences designed to enhance decision-making skills and improve team leadership and management capability. Over the 3-month period, fellows regularly convened themselves to reflect on tactics to implement new approaches and methods at their home institutions.

In the final week of the program, fellows formally presented their research project in poster format at a public scientific symposium. The graduation event typically included oral presentations by a small number of graduating fellows and by inspiring leaders in health and science from LMICs. The University of Basel subsequently awarded a University certificate and post-graduate credits using the European Credit Transfer and Accumulation System, a transferable standard for comparing study attainment and performance in higher education across Europe [20].

### Logistics and funding

The University of Basel organized and funded training events that were hosted at their campus. Novartis funded travel, accommodation, health insurance, immigration logistics, and provided a stipend. Laboratory-associated costs were borne by the Novartis hosting laboratories. Every effort was made to ensure that participation in the program was cost-neutral to the fellows and their home institutions. The program was compliant with Swiss law and ethical, legal and financial guidelines of the University of Basel and Novartis.

### Networking and post-fellowship contact

Participants were co-located in student accommodation which allowed for the development of a sense of community among fellows, provided an environment to conduct group project work in an informal setting, and helped the development of a sustainable peer social and professional network. After the fellowship, ongoing exchanges among fellows and their University of Basel and Novartis mentors were encouraged.

### Data sources and analysis methods

Demographic characteristics were extracted from fellows’ application dossiers. Fellows’ research deliverables (including research poster content and peer-reviewed journal articles) were used to summarize and categorize research activities.

Feedback was continually received over the evaluation period through spontaneous verbal and electronic communication. In addition, a global summit was convened in 2014 and attended by approximately 60 fellows who provided updates on their professional achievements at that time.

In February 2017, an on-line follow-up survey (Additional file 1) was distributed to all NGS fellows who had participated in the program from 2011 to 2016. The 2017 cohort did not participate in this survey. The survey consisted of 18 multiple-choice and open-ended questions that assessed fellows’ academic, career and professional status, and evaluated their perceptions of impact of the NGS program on their professional trajectory and achievements.

**Fig. 1:**
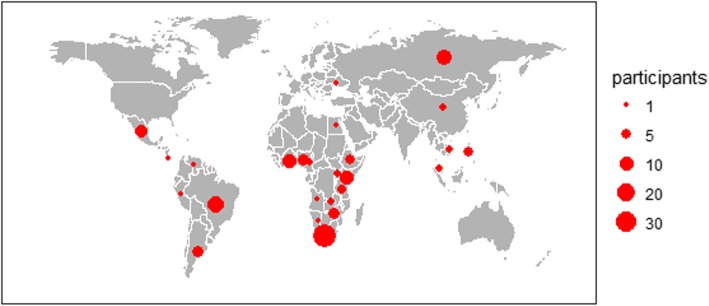
Geographic distribution of Next Generation Scientist fellows. NGS fellows were based at institutions in low- and middle-income countries in Africa, Asia, Europe, and Latin America. Since 2011, 143 fellows from 25 countries have participated. Countries that contributed the highest number of fellows were South Africa (*n* = 38) in the Africa region (62%) and Brazil (*n* = 15) in the Americas (22%)

**Fig. 2:**
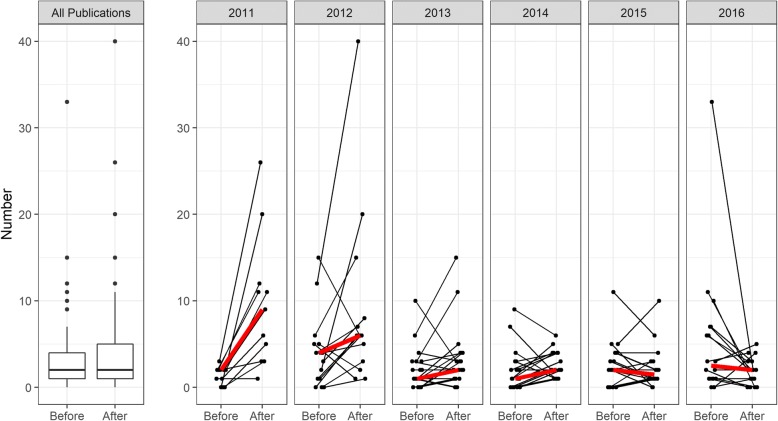
Total number of peer-reviewed publications before- and after- participation in the program. The column on the extreme left shows data for all survey respondents as a boxplot. The horizontal bar inside the box shows the median; the box encloses the inter-quartile range i.e. 50% of the data. The whiskers show the interval of values outside the box and values far outside are represented by points. The subsequent columns show before-after publication output for each survey respondent from 6 cohorts, with the thick red line showing the median for the cohort. Responses where before and after outputs were both zero have been excluded. The 2017 cohort did not participate in the follow-up survey

Data and graphical analysis were performed using RStudio (http://www.rstudio.com/), using library packages such as ggplot2 for generation of Figs. 1 and 2.

## Results

There was an increasing trend in the number of applicants to the NGS program over the duration of the program, with a notable increase after the University of Basel joined in 2014 as the co-host and accrediting academic partner (Table 1). One-hundred and forty three scientists and clinicians from LMICs took part during the 2011–2017 period reported in this paper. Demographic data and research descriptions for all fellows selected are shown in Table 2. Participants came from 73 institutions in 25 countries: Angola, Argentina, Brazil, Cameroon, China, Costa Rica, Egypt, Ethiopia, Ghana, Kenya, Malaysia, Mexico, Namibia, Nigeria, Peru, Philippines, Russia, South Africa, Tanzania, Uganda, Ukraine, Venezuela, Vietnam, Zambia and Zimbabwe (Fig. 1).

**Table 1:** Number of applicants, number selected and number of survey respondents per cohort year

Year	Applications Received^a^	Fellows Selected (%)	Respondents to 2017 Survey (%)
2011	27	15 (56)	13 (87)
2012	37	20 (54)	17 (85)
2013	108	23 (21)	23 (100)
2014	308	22 (7)	22 (100)
2015	224	20 (9)	20 (100)
2016	263	20 (8)	19 (95)
2017	219	23 (11)	n/a

^a^After removing duplicates and only retaining applications from low- and middle-income countries

**Table 2:** Demographics of participants at the start of the 3-month fellowship (*n* = 143)

General	
Gender (female)	72 (50%)
Median age (25th–75th percentile)	29 (27–33)
Region (Country)	
*Americas* (Argentina, Brazil, Costa Rica, Mexico, Peru, Venezuela)	32 (22%)
*Africa & Eastern Mediterranean* (Angola, Cameroon, Egypt, Ethiopia, Ghana, Kenya, Namibia, Nigeria, South Africa, Tanzania, Uganda, Zambia, Zimbabwe)	88 (62%)
*Western Pacific* (China, Malaysia, Philippines, Vietnam)	10 (7%)
*Europe* (Russian Federation, Ukraine)	13 (9%)
Training level	
Master’s	43 (30%)
PhD	89 (62%)
Post-doctoral	11 (8%)
Professional status^a^	
Student	88 (62%)
Public sector (including academia)	47 (33%)
Private sector (including pharma)	8 (6%)
Medical doctor	31 (22%)
Fellowship research focus^a^	
Development of a specific methodology (e.g., laboratory protocol or standard operating procedure)	111 (78%)
Training on planning or conduct of clinical trials	34 (24%)
Access to laboratory equipment, infrastructure and expertise	29 (20%)
Analysis of samples or data from the fellow’s own institution	14 (10%)
Fellowship scientific discipline	
Biological targets and pathways	32 (22%)
Chemistry, formulation, or analytical sciences	42 (29%)
Clinical research	55 (39%)
Genetics and genomics	14 (10%)

^a^Categories are not mutually exclusive

The highest proportion of fellows was based in Africa (62%). There was a predominance of doctoral or early post-doctoral scientists (70%) and an even distribution by gender (72 female and 71 male participants).

The majority (78%) of participants joined the program to learn a new research methodology for implementation back at their home institution. Others chose to develop skills relating to clinical trials; access laboratory equipment, infrastructure, and expertise; and/or use the fellowship to facilitate analyses of their own samples or data. Several fellows concentrated on skills relating to natural products, reflecting their continuing relevance to research programs in their local environments.

Fellows’ scientific focus was spread across basic research laboratories, clinical research departments, and drug formulation laboratories. Specific disease area interests were split between communicable and non-communicable diseases. The group comprised about 22% of clinician-scientists (typically MD or equivalent with MSc, PhD or Post-doc) most of whom were hosted in clinical research departments.

Follow-up data (Table 3 and Fig. 2) describing outcomes in the post-fellowship period were available for 95% of fellows from the 2011–2016 cohorts (6 fellows did not respond to the follow-up survey). Table 3 indicates that there was significant professional development as measured by progression to higher levels of trainings or employment. Most participants remained in the public sector and in academic settings in their countries of origin. Several fellows (11%) reported that they had been appointed to positions of leadership such as head of their clinical department or research unit.

**Table 3:** Professional progression of participants in the post-fellowship period (*n* = 120 at baseline; *n* = 114 at follow-up)

	Baseline (2011–2016)^a^	Follow-up (2017)
Number of NGS fellows located outside country of citizenship^a^	15 (13%)^b^	26 (23%)^c^
Highest training level		
Master’s student	35 (29%)	0 (0%)
PhD student	76 (63%)	61 (54%)
Post-doctoral	9 (8%)	53 (46%)
Employment status		
Student	71 (59%)	27 (24%)
Public sector (including academia)	43 (36%)	73 (64%)
Private sector (including pharma)	6 (5%)	14 (12%)

^a^The 2017 cohort did not participate in the follow-up survey

^b^All were pursuing post-graduate study in South Africa

^c^In post-doctoral training positions: 10 in the USA, 7 in Europe and 3 in South Africa. In full-time employment: 2 in USA and 1 in Europe and 4 in South Africa

Figure 2 illustrates the total number of manuscripts published in peer-reviewed journals before- and after participation in the fellowship as reported in the survey. These are shown for the full group of fellows and separately for each annual cohort of participants. An upward slope of the median is noted for the earlier cohorts (e.g. before 2014) and a flattening of the slope for the later cohorts, which likely reflects the longer period that the earlier cohorts had relative to the later cohorts to develop their research careers and publish peer-reviewed manuscripts.

Table 4 lists illustrative examples of program impact on fellows’ careers and contributions to various scientific disciplines. This table highlights more than 20 publications by NGS fellows and how their projects transferred value to their home institutions. No comparative control cohort data are available.

**Table 4:** Examples of NGS program outputs and outcomes

Fellowship research focus	Outputs and outcomes
Development of a methodology (e.g., laboratory protocol or standard operating procedure)	A medicinal chemistry doctoral student from Kenya improved chemical inhibition phenotyping assays used to predict drug-drug interaction potential of new molecular entities [23]; the method was subsequently transferred to the H3D Drug Discovery and Development Centre at the University of Cape Town where he was completing his PhD studies.
	A pharmacologist from Tanzania developed a rapid and reliable reversed phase high performance liquid chromatography method for simultaneous determination of selected anti-retroviral agents and lumefantrine in human plasma [24]; the method was successfully transferred to her home laboratory, facilitated completion of her PhD, and is being used by other post-graduate students at that institution researching HIV-malaria co-infection.
	A molecular biologist from Argentina developed a high-throughput screening assay for a drug target for *Brucella abortus,* the causative organism for brucellosis, a zoonotic infection that affects livestock and humans. Ten compounds with promising potency were identified after screening 44,000 compounds. This work facilitated completion of her doctoral thesis [25, 26] and formed the basis of a successful grant application at her research institute in Buenos Aires.
	A pharmacologist from Nigeria evaluated the herb-drug interaction potential of natural products in common use in his country [27, 28]. This facilitated the completion of his doctoral studies and transfer of the in vitro metabolism study methods to his institution in South Africa, and helped to enable his subsequent post-doctoral and faculty appointment.
	A cellular and molecular biologist from Brazil conducted laboratory studies to develop mechanistic understanding of cell surface immune responses of helper T cells. This facilitated continuation of his doctoral studies on Chagas disease [29]. The methodologies were used by his home laboratory to conduct in vitro assays to examine the role of the different T-cell subsets and cytokines in disease progression.
Training on planning or conduct of clinical trials	A medical doctor from South Africa obtained practical skills in operational planning and execution of first-in-human (FIH) studies [14] that were subsequently applied to a FIH study of a malaria drug candidate discovered at H3D in partnership with Medicines for Malaria Venture [30]. She continues with an active clinical pharmacology research agenda including studies to understand pharmacogenetics and clinical response [31].
	A medical oncologist from Brazil worked with an early clinical development team to learn procedures relating to trial protocol amendments. This facilitated direct interaction with in-house experts for input into her doctoral studies [32–35]. Upon return to her clinic in Brazil, she continued as clinical investigator on multiple oncology translational medicine clinical studies.
	A medical doctor from Ethiopia compared mechanistic explanations of drug induced liver injury across multiple publicly available clinical candidates [36]. He also acquired clinical trial skills and networks to establish a clinical pharmacology unit [14] after his return to Ethiopia, and has an active senior role in multiple aspects of clinical research.
Access to laboratory equipment, infrastructure and expertise	A geneticist from South Africa documented genetic diversity in Black South Africans from Soweto, learned bioinformatics techniques, and constructed a large database of African genetic diversity for further analyses and training purposes [37].
	A geneticist from South Africa identified a novel mutation in the CHST6 gene as a cause of macular corneal dystrophy in a Black South African family, which was used for genetic counselling of the family [38].
	A drug formulation scientist from Kenya assessed alternate liposomal parenteral formulations to solubilize poorly soluble drug substances while working with the nano-technology unit. He applied these technologies to potential anti-malarial drug formulations [39, 40] as part of his doctoral studies. He currently collaborates with South African scientists and their team has been successful in grant applications relating to malaria research.
Analysis of samples or data from the fellow’s own institution	A medicinal chemist from Kenya evaluated the metabolism and pharmacokinetics for a series of new deoxyamodiaquine-based compounds. This work was directly applied to the drug discovery program at H3D [41].
	A pharmacognocist/phytochemist from Ghana assessed natural products to demonstrate anti-plasmodial and medicinal potential [42, 43]. These initial studies played a key role in the expansion of the natural products research laboratory at his home institution.

The follow-up survey revealed an enduring network after completion of the 3-month fellowship. Nearly two-thirds of participants (64%) reported ongoing regular contact with other fellows and 35% reported sporadic contact. Only 3 fellows reported having no continuing contact with their peers. Ongoing contact was also observed between fellows and their mentors at University of Basel or Novartis; 86% of fellows reported regular or sporadic contact. Active collaboration, as defined by ongoing project-related interactions, was reported by 8% of fellows for peer-to-peer partnerships and by 12% of fellows for fellow-to-mentor partnerships.

In open-ended comments on the follow-up survey, NGS fellows reported concrete scientific benefits such as access to equipment and data; expediting completion of post-graduate qualification; and “soft” benefits including networking, reputation, and broadened perspectives on work ethic and culture. Fellows also reported improvements in research productivity via publications, conference presentations, success with funding applications and involvement in clinical trials. As a complement to individual scientific research development, the respondents also provided examples where they themselves transferred skills to colleagues at home. Table 5 provides a summary of the benefits of the program as reported by NGS fellows and their mentors.

**Table 5:** Program benefits reported by NGS fellows, home institution supervisors and host institution mentors

Fellows and supervisors	Mentors
Access to data, biomedical technologies, and industry scientific expertise	Insights into local health care system and infrastructure in low and middle income countries (LMICs)
Enhanced skills to formulate relevant and impactful research questions	Insights into differences in disease manifestation and patient needs between LMICs and high-income countries (HICs)
Opportunities for networking and collaboration (e.g., with NGS fellows, industry colleagues, academic collaborators through networks created during the fellowship)	Development of collaborative relationships with local academic centers with similar or complementary research interests
Expedited completion of post-graduate qualification	Contribution to a social responsibility-driven mission
Improved understanding of career opportunities in industry and academia	
Development of local scientists and heightened global awareness of research environment	
Increased professional confidence, success in securing employment and leadership positions	

## Discussion

A 7-year structured, immersive mentorship program contributed to professional development for a carefully selected group of 143 scientists and clinicians from 25 LMICs. A unique feature of the program was the bringing together of cohorts of researchers at the launch stage of their scientific careers. This appears to have facilitated a sustained network of scientists in LMICs despite the relatively short 3-month duration of the on-site component. The governance of the NGS program was also novel, with voluntary shared responsibility by a range of academic, public, and private partners. Understanding key enablers of the program, and its limitations up until now, is fundamental to improving the program in the future and may inform the design and implementation of similar capacity-building efforts in LMICs.

A widely acknowledged success that was highlighted by fellows and mentors was the opportunity for mutual learning in topic areas that would not easily be attained through other mechanisms. By design, fellows benefited from specific scientific and professional development training. In many cases, mentors similarly benefited from the knowledge exchange (e.g., relating to natural products; pharmacogenetics in diverse populations; and first-hand accounts of health priorities). In nearly all cases, mentors stated that the lessons they learned about patients, diseases, and health systems in LMICs broadened their perspectives about science and medicine globally in ways that influenced their own professional and personal activities. This gives credence to the Nigel Crisp assertion that “rich countries can learn a great deal about health and health services from poorer ones and that combining the learnings from rich and poor countries can give us new insights into how to improve health” [18]. We suspect that the reciprocated respect that developed between fellows and mentors because of shared learning experiences played an important role in paving the way for continued joint collaborations in the post-fellowship period.

Another program enabler was the flexibility associated with fellows’ research curricula. Fellows were not pigeon-holed towards pre-determined activities—rather, research programs were individualized according to the needs of each fellow, agreement between fellows and mentors, and relevance to priorities at fellows’ home institutions and countries. The hosting laboratories at Novartis needed to be able to support the science that the fellow would conduct, but the fellow’s research program did not need to directly advance the research agenda of the hosting laboratory. Providing support for fellows’ research interests was not always entirely straightforward. For example, some projects involved analysis of plant or biological samples from fellows’ home institutions, which required considerable effort by both the home and host institutions to ensure compliance with the ethical-legal regulations associated with cross-border transfer of materials. The benefit, however, was scientific progress and attainment of valuable experience on both sides for implementing globally-focused scientific research. For all projects, research success was a result of thorough planning before the fellowship and on-going interactions afterwards. It was felt that tailoring activities to the needs of fellows was a significant factor in the impressive scientific productivity that resulted (for example see the peer-reviewed publications cited in Table 4) despite the short 3-month duration of the on-site phase.

The high retention of fellows in their home countries (> 75%) and employment in the public or academic sector is noteworthy - only 4 scientists reported working for a pharmaceutical company after completion of their studies in contrast to over 60% being employed in the public or academic sector (Table 3). Although we acknowledge that private sector employment for highly trained researchers is scarce in LMICs, we were nevertheless encouraged by this result, given valid concerns that inviting high caliber scientists into well-resourced research environments might further contribute to the “brain drain” from these countries [4–6]. Factors related to this success likely include: an unambiguously stated goal of the program for local capacity development rather than staff recruitment; a rigorous selection process capable of identifying candidates with a desire to contribute to their local healthcare research; and the short 3-month on-site stay. In some cases, this was assisted by strong links with home supervisors who provided an enabling home base to return to and continue on a rejuvenated career trajectory.

The NGS program’s multi-sectoral academic-public-private partnership model worked well. Involvement of the University of Basel as a coordinating entity was instrumental in providing academic credibility, and the collaborative framework that developed with science as an organizing principle helped the industry partner to be viewed as an equal contributor rather than simply a funding source. Within LMICs, the program often served as a complement to locally funded government or non-governmental capacity-building initiatives and in some instances stimulated the establishment of new partnership programs.

Much has been written to acknowledge the secondary effects of strengthening research capacity on health in under-resourced environments. These viewpoint and perspectives papers typically highlight existing programs and advocate for new collaborative efforts involving local and international governments, private sector groups and large funding bodies [2, 3]. The program described here has similarities in its genesis and goals with the Career Development Fellowship (CDF), jointly administered by the World Health Organization’s Special Programme for Research and Training in Tropical Diseases (WHO/TDR) and the European & Developing Countries Clinical Trials Partnership (EDCTP) [7]. The CDF was founded as a partnership involving a pharmaceutical company, GlaxoSmithKline Biologicals, and has been independently evaluated as being relevant, efficient and effective with potential for impact and sustainability. However despite expansion to include multiple partners, the number of participants reported in the evaluation remain modest [7], which we believe underscores the need for more programs such as these.

Future priorities for the NGS program will be to increase involvement of the home academic supervisors. Our experience has been that home supervisor involvement leads to more successful project outcomes, whereas a lack of involvement could make the reintegration to the home laboratories more difficult. Fellows have highlighted that whilst techniques are relatively easy to transfer back, research culture traits can be more difficult due to a confounding interplay of culture and infra-structure deficits between research sites located in high-income countries and the academic centers in LMIC settings. Closer engagement with the home mentors and institutions might represent one component of a solution. Ideally this would be coupled with enriched and expanded post-fellowship activities. There has been substantial interest from NGS fellows in formalizing post-fellowship interactions, in part due to recognition of the benefits that could be realized by capitalizing on their large, growing network of emerging scientific leaders in LMICs. Preferably, this will be accomplished by putting primary governance of the activities with the fellows themselves. As with many capacity-building efforts, we are currently faced with the challenge of identifying methods for scaling up the program while maintaining the individualized approach that is thought to be crucial to the program’s success. We have initiated exploratory high-level discussions with several major research funding organizations and are hopeful that the program can be grown in future program cycles.

The above benefits described notwithstanding, we acknowledge that there are limitations to capacity-building via the approach described, and there were also limitations to our ability to evaluate the program. As with all survey-type methodologies there is the possibility of reporting bias, though we took the exceedingly high response rate (ranging 87–100% across the 7 cohorts of participants) as a sign that reporting bias in this evaluation was likely to be minimal. To mitigate potential bias by the program funders (Novartis and the University of Basel) in the evaluation of the program, we assembled a multi-sectoral public-academic-private team to conduct the study reported here. This team included some supervisors of NGS fellows from their home institutions and were thus in a position to contextualise the overall survey data against their own personal impressions of the impact of the program on their students. The core component of the NGS program is a 3-month intensive mentored research experience, and there are natural confines to the extent of professional development that can be achieved in this time-limited period. While for many participants the core program set into motion long-term collaborative activities (which was encouraged), these activities were not supported with dedicated resources. Another potential inherent weakness with the program design was mentoring of fellows outside of their home institution. This required added financial resources and logistical considerations, and temporarily removed scientists from the precise environments where capacity-building is needed. However, the rationale was to enable sufficient exposure to new resources (e.g., other scientists, laboratories, tools, and technologies) that would enrich their professional development; in addition, this approach served to facilitate peer-to-peer networks that would likely not have been established to the same degree without bringing fellows together face-to-face. Quantifying and valuing the multiple hard and soft dimensions of long-term impact of capacity development programs remain challenging. Logistical impracticalities preclude the conduct of a controlled study – we propose the development of methods to quantify the social impact of capacity development programs in monetary terms, similar to the valuation of an environmental footprint [21, 22]. The current evaluation may have been confounded because the NGS program application process favored selection of high-performing candidates, and because fellows were unable to keep responses in the online follow-up survey confidential from the program organizers who administered the survey. Nevertheless, we believe that the very high response rate to the follow-up survey, which was conducted up to 6 years after participation, coupled with meaningful self-reported outputs, is an indicator that many fellows felt that the NGS program had tangible benefits to their professional development.

## Conclusions

This 7-year structured, immersive mentorship program with 143 scientists and clinicians from 25 LMICs provides an example for how multi-sectoral partners can contribute to scientific and professional development of researchers in LMICs. Our experience substantiates the view that capacity-building efforts in LMICs should be tailored to the specific needs of beneficiaries to be maximally effective. Lessons learned may be applied to the design and conduct of other programs to strengthen science ecosystems in LMICs.

## Additional file

Additional file 1: NGS Survey Instrument. (PDF 156 kb)

## Abbreviations

LMIC: Low- and middle- income countries
NGS: Next Generation Scientist
