# Supplementary material for: The Next Generation Scientist program: capacity-building for future scientific leaders in low- and middle-income countries
Source: BMC Med Educ. 2018 Oct 10;18:233. doi: 10.1186/s12909-018-1331-y (PMC6180641; doi:10.1186/s12909-018-1331-y)
Supplement: Supplementary file 1 — NGS Survey Instrument. (PDF 156 kb) [file 12909_2018_1331_MOESM1_ESM.pdf]

Dear NGS Fellow,

We recently shared a link to the poster that was presented on the NGS program. Take a look at https://goo.gl/juvLx5. We are thrilled that you are 1 of more than 120 scientists from 24 countries across the world who is a graduate, and hence a member of this family. We are currently preparing a more comprehensive manuscript on the impact of the program that will be submitted as a peer-reviewed publication together with academic co-authors including your co-hosts, the University of Basel.

We would appreciate a few minutes of your time (<10 minutes) in order to provide feedback on the impact that the program has had on your scientific and professional career.

The purpose of presenting this in a peer-reviewed publication is to report the impact of the program and to advocate for wider implementation of programs such as NGS to develop the next generation of researchers who could contribute to locally relevant solutions to healthcare challenges that exist in low and middle income countries. For the most part, the data from this survey will be presented in summary form in tables and figures. There will also be a limited number of case-studies that could illustrate program impact. If chosen, please be assured that we will seek your review and approval before identifying you.

Thank you in advance for your prompt response to the survey.

If you have any questions, please contact Colin Pillai by email or telephone.
goonaseelan.pillai@novartis.com
+41795976335

Warm regards,

Colin Pillai PhD
Head: Global Scientific Capabilities CoE
Novartis Pharma AG
CH-4002 Basel, Switzerland

PART 1 - General Information

1. Name

Your name will help us link to other data. Please note that most results will be reported in composite form as shown in the poster (https://goo.gl/juvLx5). We will not reveal your identity without your permission.

* 2. Year of participation in the NGS program

- ○ 2011
- ○ 2012
- ○ 2013
- ○ 2014
- ○ 2015
- ○ 2016

* 3. Background

Current institution

Location (City, Country)

* 4. What was your qualification (completed degree) at time of attending NGS?

- ☐ PhD
- ☐ Master
- ☐ Medical Doctor
- ☐ Undergraduate
- ☐ Other (please specify)

* 5. What is your current highest qualification?

- [ ] PhD
- [ ] Masters
- [ ] Medical Doctor
- [ ] Undergraduate
- [ ] Other (please specify)

* 6. What was your role at time of attending NGS?

- [ ] Student
- [ ] Post-doc
- [ ] Head of Unit
- [ ] Employed in Private Sector e.g. industry
- [ ] Employed in Public Sector e.g. university
- [ ] Employed in Government
- [ ] Other (please specify)

* 7. What is your current role?

- [ ] Student
- [ ] Post-doc
- [ ] Head of Unit
- [ ] Employed in Private Sector e.g. industry
- [ ] Employed in Public Sector e.g. university
- [ ] Employed in Government
- [ ] Other (please specify)

* 8. What was your original field of training?

- ○ Pharmacy
- ○ Medicine
- ○ Quantitative Sciences e.g. Statistics/Mathematics/Engineering
- ○ Biological Sciences e.g. laboratory sciences, target identification
- ○ Chemistry-related Sciences e.g. chemical discovery, drug formulations, analytics
- ○ Genetics/Genomics
- ○ Other (please specify)

* 9. What is your current scientific discipline?

If "other" please explain.

- ○ Medical Doctor
- ○ Quantitative Scientist e.g. statistician, modeler, pharmacometrician
- ○ Laboratory Scientist
- ○ Clinical Trialist
- ○ Other (please specify)

* 10. What was your therapeutic area interest at the time of attending NGS?

- [ ] Communicable Diseases
- [ ] Non-Communicable Diseases
- [ ] Not-Applicable
- [ ] Both Communicable and Non-Communicable Diseases

Please state the therapeutic area using 1-2 key words e.g. tuberculosis. If you answered not-applicable, please explain e.g. "My work was methodological"

* 11. What is your current therapeutic area interest?

- [ ] Communicable Diseases
- [ ] Non-Communicable Diseases
- [ ] Not-Applicable
- [ ] Both Communicable and Non-Communicable Diseases

Please state the therapeutic area using 1-2 key words e.g. tuberculosis. If you answered not-applicable, please explain e.g. "My work was methodological"

* 12. Are you still in contact with one or more Novartis or University of Basel colleagues?

- [ ] Regular basis
- [ ] Collaboration for a specific project
- [ ] Sporadically
- [ ] No - lost contact
- [ ] Explain (optional)

* 13. Are you still in contact with one or more NGS fellows - either from your original group or another cohort?

- [ ] Regular basis
- [ ] Collaboration for a specific project
- [ ] Sporadically
- [ ] No - lost contact
- [ ] Explain (optional)

PART 2 - Assesment of NGS Impact

Please note that questions 14, 15 and 16 are linked (before, after and your assessment of how much the program contributed to the achievement)

* 14. BEFORE the NGS program, how many...

...publications have you submitted ?

...publications related to your NGS research project have you submitted ?

...conference presentations (oral or poster) have you submitted ?

...training courses (including laboratory skills) did you run or contribute to ?

...post-graduate students did you supervise ?

...research grants have you submitted ?

...clinical trials have you been involved with (as a researcher)?

* 15. AFTER the NGS program, how many...

...publications have you submitted ?

...publications related to your NGS research project have you submitted ?

...conference presentations (oral or poster) have you submitted ?

...training courses (including laboratory skills) did you run or contribute to ?

...post-graduate students did you supervise ?

...research grants have you submitted ?

...clinical trials have you been involved with (as a researcher)?

* 16. We acknowledge that multiple factors could have contributed to your achievements above. Therefore please rate how relevant the NGS program was in preparing you for the achievements listed above, on a scale from 1 (not at all relevant) to 5 (extremely relevant). By relevance, we mean how much this contributed to your achievement. The more you feel the program was relevant for the specific output you've reported, the higher you would rate it. The less you feel the the program was relevant, the lower you would rate it.

	1-Not at all relevant	2	3	4	5-Extremely relevant	Not Applicable
Training Courses that you contributed to/ran	○	○	○	○	○	○
General Publications	○	○	○	○	○	○
Publications related to NGS research project	○	○	○	○	○	○
Poster Presentations	○	○	○	○	○	○
Oral Presentations	○	○	○	○	○	○
Grants	○	○	○	○	○	○
Post-Graduate Students Supervised	○	○	○	○	○	○
Clinical Trials	○	○	○	○	○	○

* 17. Were the European Credit Transfer System (ECTS) points from University of Basel useful?

- ◯ I did not receive ECTS points
- ◯ I received ECTS points and have not used them thus far
- ◯ I received ECTS points and found them useful. Please explain below

18. Please add any other evidence of impact of the NGS program that you think might be of relevance e.g. add a list of publication citations.

PART 3 - Next Steps

* 19. Would you agree to being identified as an example of program impact ? Please note that we will first share specific details for your review and approval if we choose to list you as a case example.

- ◯ Yes
- ◯ No

20. Are there ways that the NGS program benefited your scientific or professional career that have not been reflected in this survey?”

21. Do you have any other comments, questions, or concerns?

Thank you very much for taking the time to participate in this survey.
